# Crystal Structures of Leukotriene C_4_ Synthase in Complex with Product Analogs: IMPLICATIONS FOR THE ENZYME MECHANISM*

**DOI:** 10.1074/jbc.M113.534628

**Published:** 2013-12-23

**Authors:** Damian Niegowski, Thea Kleinschmidt, Ulrika Olsson, Shabbir Ahmad, Agnes Rinaldo-Matthis, Jesper Z. Haeggström

**Affiliations:** From the Division of Chemistry II, Department of Medical Biochemistry and Biophysics, Karolinska Institutet, S-107 77 Stockholm, Sweden

**Keywords:** Asthma, Glutathion, Leukotriene, Lipid-binding Protein, Membrane Proteins, LTA4, LTC4, LTC4 Synthase

## Abstract

Leukotriene (LT) C_4_ synthase (LTC4S) catalyzes the conjugation of the fatty acid LTA_4_ with the tripeptide GSH to produce LTC_4_, the parent compound of the cysteinyl leukotrienes, important mediators of asthma. Here we mutated Trp-116 in human LTC4S, a residue proposed to play a key role in substrate binding, into an Ala or Phe. Biochemical and structural characterization of these mutants along with crystal structures of the wild type and mutated enzymes in complex with three product analogs, viz. *S*-hexyl-, 4-phenyl-butyl-, and 2-hydroxy-4-phenyl-butyl-glutathione, provide new insights to binding of substrates and product, identify a new conformation of the GSH moiety at the active site, and suggest a route for product release, aided by Trp-116.

## Teaser

**Background:** Leukotriene (LT) C_4_ synthase (LTC4S) is a membrane protein catalyzing the formation of pathogenic mediators of asthma.

**Results:** Crystal structures of complexes of WT and mutated LTC4S with three product analogs.

**Conclusion:** Evidence for the binding mode of LTA_4_ and LTC4 at the active site of LTC4S and a mechanistic model.

**Significance:** New insights into the molecular mechanism of LTC4S.

## Introduction

The leukotrienes are proinflammatory lipid mediators formed along the 5-lipoxygenase pathway of arachidonic acid metabolism (1). In this cascade, leukotriene (LT)^4^ C_4_ synthase (EC 4.4.1.20) catalyzes conjugation of the unstable epoxide intermediate LTA_4_ ((5*S*)-*trans*-5,6-oxido-7,9-*trans*-11,14-*cis*-eicosatetraenoic acid) with GSH to form LTC_4_ ((5*S*)-hydroxy-(6*R*)-*S*-glutathionyl-7,9-*trans*-11,14-*cis*-eicosatetraenoic acid), the parent compound of the cysteinyl leukotrienes C_4_, D_4_, and E_4_. These three compounds together comprise the classical slow reacting substance of anaphylaxis, an established mediator of human asthma (2, 3). Cysteinyl leukotrienes signal via two G-protein coupled receptors denoted CysLT1 and CysLT2 and antagonists of the former receptor are used in the clinical management of asthma (4–6). Unfortunately, ∼40% of all patients do not respond to this treatment (7). Therefore, there is a need for more efficient therapeutic agents. Because recent data indicate that at least five different GPCR can transmit cysteinyl leukotriene signaling, the biosynthetic enzyme LTC4S has emerged as a promising target to eliminate mediator production and bioactions (8).

**FIGURE 1.**
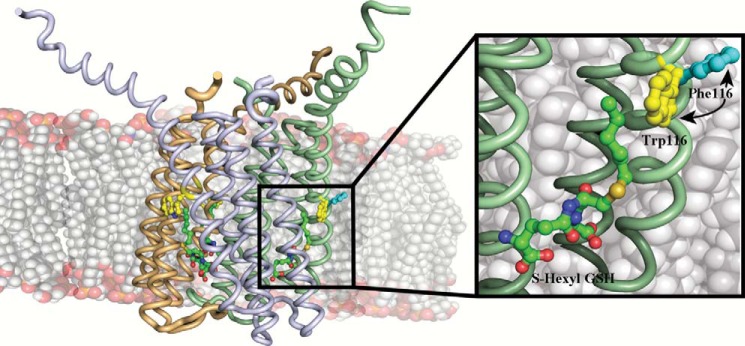
**Side view of the LTC4S trimer showing the three monomers in *pale green*, *beige*, and *light gray*.** Analog I, Trp-116, and Phe-116 (from the structure of W116F) are depicted as balls and sticks. *Inset*, close up of analog I binding in the active site showing the Phe in the W116F mutant, which has rotated outwards and into the membrane.

LTC4S is a trimeric integral membrane protein (Fig. 1) and a member of the MAPEG (membrane-associated proteins in eicosanoid and glutathione metabolism) family, which also includes three microsomal GSH S-transferases, 5-lipoxygenase activating protein, and microsomal prostaglandin E synthase 1 (9, 10).

Integral membrane proteins are essential in all living organisms for the shuttling of metabolites across lipid membranes as well as catalyzing enzymatic reactions. Knowledge of their structures, functions, and mechanisms is severely lacking compared with their soluble counterparts (Protein Data Bank) (12, 13). In particular, very little is known about the selectivity and specificity of proteins utilizing lipids as substrates within a lipid membrane. LTC4S and most MAPEG family members carry out their function in the interior of the lipid membrane and apart from having important interactions with constituents of the bilayer, which presumably are needed for stability and conformation, and they are also able to distinguish these lipid species from their *in vivo* substrates.

**FIGURE 2.**
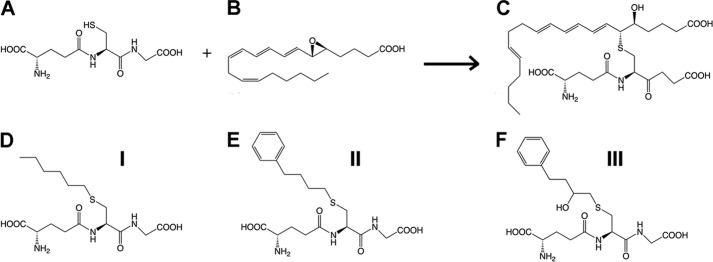
*Top row*, the LTC4S conjugation reaction between GSH (*A*) and LTA_4_ (*B*), and product LTC_4_ (*C*). *Bottom row*, comparison of analogs I (*D*), II (*E*), and III (*F*).

The crystal structure of human LTC4S has been solved and identified a unique horseshoe-shaped conformation of GSH bound at the active site by residues from two neighboring monomers (14, 15). It was postulated that the lipid substrate LTA_4_ binds in a hydrophobic crevice between two monomers and that Trp-116 could act as a “molecular ruler” for binding of LTA_4_ with its ω-end buried under the aromatic side chain. Furthermore, mutational and spectroscopic studies have identified Arg-104 as the residue responsible for thiol activation (8, 16). In the present study, we explored the functional role of Trp-116 in human LTC4S by mutational analysis and crystallography. As a surrogate for the labile endogenous leukotrienes (see Fig. 2), we synthesized three stable product analogs, viz. hexyl-, 4-phenyl-butyl-, and 2-hydroxy-4-phenyl-butyl-glutathione, which thus carry a lipid tail to mimic the fatty acid backbone of LTA_4_. We then determined the crystal structures of LTC4S in complex with these compounds. Together, our results offer new insights to binding of LTA_4_ at the active site and allow us to propose a model for product release.

## EXPERIMENTAL PROCEDURES

### Materials

Imidazole, Tris base, NaCl, KCl, Triton X-100, sodium deoxycholate, *S*-hexylglutathione-agarose, probenecid, GSH, and 2-mercaptoethanol were obtained from Sigma. Platinum *Pfx* DNA polymerase and deoxyribonucleotides were from Invitrogen. Dodecyl maltoside was obtained from Anatrace. LTA_4_ methyl ester (BIOMOL) in tetrahydrofuran was saponified with 1 m LiOH (6%, v/v) for 48 h at 4 °C. All other chemicals were obtained from common commercial sources.

### Site-directed Mutagenesis

Site-directed mutagenesis was carried out according to the QuikChange protocol (Stratagene, La Jolla, CA) essentially as described (8). The primers for the W116A mutant were as follows: 5′-GCG GCG CGC GCC CTC GCG CTG CTG GTG GCG-3′ (forward) and 5′-CGC CAC CAG CAG CGC GAG GGC GCG CGC CGC-3′ (reverse). For W116F, the primers were as follows: 5′-GCG GCG CGC GCC CTC TTC CTG CTG GTG GCG-3′ (forward) and 5′-CGC CAC CAG CAG GAA GAG GGC GCG CGC CGC-3′ (reverse). The integrity of the protein and the mutations were verified by DNA sequencing (SEQLAB, Göttingen, Germany).

### Protein Expression and Purification

Wild type and mutated LTC4S proteins were expressed in *Pichia pastoris* and purified with two steps of affinity chromatography essentially as described (15). For crystallization purposes, proteins were subjected to a buffer exchange on Superdex 200 16/60 (GE Healthcare) equilibrated with 25 mm Tris-HCl (pH 7.2), 0.03% (w/v) dodecyl maltoside, and 300 mm NaCl. Chromatographic fractions containing WT enzyme, W116A, and W116F LTC4S were concentrated to 1–3 mg/ml by ultrafiltration. Protein concentrations were determined according to the method of Lowry (Sigma) with BSA as a standard. SDS-PAGE was performed on a PhastSystem (GE Healthcare) utilizing 10–15% gradient gels. Protein bands were visualized with Coomassie Brilliant Blue.

### Synthesis of Substrate Analogs

#### 

##### Analog I, ((S)-2-amino-5-((R)-1-(carboxymethylamino)-3-(hexylthio)-1-oxopropan-2-ylamino)-5-oxopentanoic acid)

A suspension of reduced l-glutathione (3 g, 9.8 mmol) and 20 ml of 1 m NaOH was prepared using a magnetic stirrer at room temperature. About 60 ml of 95% EtOH was added to the solution in small portions, until the “cloud point” was reached. 1-Iodohexane (1.5 ml, 10 mmol) was added, and the reaction was stirred at room temperature overnight. Next, the pH was adjusted to 3.5 with 67% hydriodic acid and the reaction mixture was cooled on an ice bath for a few hours, and a precipitation was formed, which was filtered off and washed with 10 ml of 95% EtOH and 100 ml cold water. The isolated precipitation was dried in vacuum to yield 2.6 g (6.6 mmol, 67%) of *S*-hexylglutathione as a white/light yellow powder.

##### Analog II, ((S)-2-amino-5-((R)-1-(carboxymethylamino)-1-oxo-3-(4-phenylbutylthio)propan-2-ylamino)-5-oxopentanoic acid)

4-Phenylbutan-1-ol (0.5 ml, 3.3 mmol), triphenylphosphine (1.15 g, 4.4 mmol), imidazole (0.31 g, 4.6 mmol), and iodine (1.14 g, 4.5 mmol) were dissolved in 100 ml Et_2_O/MeCN 2:1 in a round bottom flask and then bubbled with Ar for a few minutes. The reaction was stirred at room temperature and followed by TLC toluene:EtOAc 6:1, showing complete conversion of starting material to product after 2.5 h. The reaction mixture was then washed with 3 × 50 ml H_2_O, 2 × 50 ml saturated Na_2_S_2_O_3_ and 2 × 50 ml saturated NaCl. The organic layer were dried with MgSO_4_ (s), filtered, and evaporated to give 0.55 g (2.1 mmol, 64%) 4-iodobutylbenzene as a white powder.

Reduced l-glutathione (0.37 g, 1.2 mmol) was dissolved in 2 ml 1 m NaOH. About 4.5 ml of 95% EtOH was added dropwise until the cloud point was reached. 0.32 g (1.2 mmol) of 4-iodobutylbenzene was added and the reaction was stirred at room temperature over night. Hydroiodic acid was added dropwise until pH reached 3.5 and then cooled at 8 °C. The product mixture was kept at 8 °C to allow precipitation. The precipitated product was filtered and washed with water and EtOH and then dried under vacuum. The product was isolated as a white powder at a yield of 23% (0.13 g).

##### Analog III, ((2S)-2-amino-5-((2R)-1-(carboxymethylamino)-3-(2-hydroxy-4-phenylbutylthio)-1-oxopropan-2-ylamino)-5-oxopentanoic acid)

4-Phenyl-1-butene (1.3 ml, 8.7 mmol) was diluted in 10 ml dichloromethane at 0 °C. 3-Chloroperbenzoic acid (1.5 g, 8.7 mmol) was added in one portion, and the reaction mixture was stirred overnight without further cooling. The reaction was quenched and washed with 2 × 20 ml saturated NaHCO_3_ and further washed with by 2 × 20 ml water. The organic layer was dried with MgSO_4_ (s), filtered and, evaporated. The product was purified on a silica column giving 0.69 g (4.7 mmol, 54%) of (*S*)-2-phenethyloxirane as a white powder.

A suspension of reduced l-glutathione (2.13 g, 6.9 mmol) and 20 ml MeOH:triethylamine 4:1 was bubbled with Ar for 5 min. (*S*)-2-phenethyloxirane (0.68 g, 4.62 mmol) was added in one portion. The reaction mixture was then put in the oven at 37 °C for 24 h. The product mixture was cooled to room temperature and evaporated. The resulting white powder was dissolved in cold water and adjusted to pH 3 with 6 m HCl. The cloudy product suspension was then put in the fridge for 3 days to enable precipitation of the product. The product was filtered, washed with cold water, and dried under vacuum to give 0.87 g (1.92 mmol, 42%) of white crystals. All analogs were verified for the correct mass by electrospray ionization MS and fragmented by collision-induced dissociation.

### Enzyme Kinetics

Enzyme activity toward GSH and LTA_4_ for wild-type, W116F, and W116A LTC4S was determined with aliquots of enzyme (0.1 μg for GSH and 0.05 μg for LTA_4_) diluted to 100 μl with 25 mm Tris-HCl (pH 7.8) supplemented with 0.05% Triton X-100. When kinetic parameters for GSH and LTA_4_ were determined, the buffer was supplemented with 36 μm of LTA_4_ and 5 mm of GSH, respectively. The incubations were performed at room temperature and stopped after 15 s by the addition of 200 μl of methanol followed by 100 μl of water. Prostaglandin B2 (620 pmol) was added as an internal standard before reverse phase HPLC, which was performed on a Waters Nova-Pak C18 column (3.9 × 150 mm) eluted with a mixture of acetonitrile/methanol/water/acetic acid (30:20:50:0.1, v/v) at an apparent pH 5.6 and at a flow rate of 0.8 ml/min (LKB 2150 pump). Qualitative analysis was performed by comparison with the retention time of synthetic LTC_4_ using a Milton Roy SpectroMonitor 3100 detector. The amount of LTC_4_ was quantified by calculating the ratio of the peak area compared with the internal standard prostaglandin B2. The *k*_cat_ and *K_m_* (Table 1) values were determined from the initial velocity of the LTC4S-catalyzed reaction measured as a function of substrate (GSH or LTA_4_) concentrations. The initial velocity data were fitted to the Michaelis-Menten equation using GraphPad Prism. The *k*_cat_ value was calculated from the *V*_max_ and enzyme concentration ([E]) according to the equation, *k*_cat_ = *V*_max_/[E] (see Table 1). For determination of IC_50_ values of analogs I–III against WT or mutated LTC4S, aliquots of LTC4S (0.04–0.1 μg) were diluted to 100 μl with 25 mm Tris-HCl (pH 7.8), 0.05% Triton X-100, and 5 mm GSH and incubated with 30 μm LTA_4_. Experimental data were fitted with the Michaelis-Menten equation using GraphPad Prism and RFFIT in SIMFIT to calculate the kinetic parameters *k*_cat_, *K_m_*, and *k*_cat_/*K_m_* (SIMFIT software).

**TABLE 1:** **Kinetic parameters for WT and mutated LTC4S against GSH and LTA_4_**

	*k*_cat_	*K_m_*	*k*_cat_/*K_m_*
	*s*^−*1*^	μ*m*	*m*^−*1*^/*s*^−*1*^
WT-GSH	12 ± 0.7	300.0 ± 60	4 ± 1.1 × 10^4^
W116F-GSH	17.7 ± 1.3	2380 ± 440.0	0.7 ± 0.07 × 10^4^
W116A-GSH	16.8 ± 1.2	1420 ± 290.0	1.1 ± 0.1 × 10^4^
WT-LTA_4_	26 ± 4	30.0 ± 10	8.7 ± 4 × 10^5^
W116F-LTA_4_	74.3 ± 12.5	94.6 ± 29.4	7.3 ± 0.8 × 10^5^
W116A-LTA_4_	32 ± 2.7	37.9 ± 8.69	8.1 ± 0.98 × 10^5^

### Crystallization

The crystals for the WT enzyme, W116F, and W116A LTC4S were obtained using sitting drop vapor diffusion at room temperature at a concentration of 3.5 mg/ml. 1 μl of protein solution supplemented with 1 mm GSH were mixed with 1 μl of reservoir solution containing 1.8–2.2 m NH_4_SO_4_, 0.2 m NaCl, and 0.1 m sodium cacodylate, pH 6.1–6.8. Crystals were obtained after 1 day at room temperature and matured in size within 4 days. Soaks were conducted in the reservoir solution with the addition of 1 mm hexyl-GSH in time intervals ranging from 30 s to 24 h. The crystals were cryo-protected with the addition of 15% (v/v) glycerol in the presence of 1 mm analog I in the reservoir solution after which they were flash frozen in liquid nitrogen. For analogs II and III, a co-crystallization experiment was conducted. Protein was concentrated on an Amicon® Ultra-10 filter (Millipore) and then 1 mm of analog was added and incubated for 20 min. After a quick high speed spin the protein was subjected to crystallization trials.

### Data Processing and Refinement

Data were collected at the Helmholtz-Zentrum Berlin, BESSY beamline 14.1 (W116A mutant), the European Synchrotron Facility beamline ID14.4 (W116F mutant and LTC4S in complex with analog II and III), and the European Synchrotron Facility beamline 23.2 (LTC4S-analog I complex). The data were processed using XDS and scaled with SCALA (18, 19). Data cut-off was chosen with the new assessment criteria based on ½ CC (20). It is worth mentioning that even when employing the classical criteria for data cut-off of *I*/σ and *R*_merge_, the resolution was only marginally worse, and little or no changes were observed in the electron density maps. The structure was solved using molecular replacement with PHASER using a modified PDB code 2UUI with waters and lipids removed (21). Refinement and simulated annealing was carried out with REFMAC and the PHENIX suite (22, 23). To remove possible model bias, 25 macrocycles of simulated annealing were carried out prior to model building and ligand introduction with a starting temperature of 5000 K. Model building was done using Coot (24). The analogs were identified from the original maps and also confirmed using *F_o_* − *F_o_* maps calculated with FFT from PDB codes 2UUI, 2UUH, and 3PCV (data not shown). All figures were produced using PyMOL software (25). X-ray statistics are presented in Table 2.

**TABLE 2:** **X-ray data collection and refinement statistics** Values in parentheses are for highest-resolution shell. r.m.s., root mean square.

	WT, analog I	W116A, analog I	W116F, analog I	WT, analog II	WT, analog III
**Data collection**					
Space group	F23	F23	F23	F23	F23
Cell dimensions	*a* = 170.3, *b* = 170.3, and *c* = 170.3 Å; α=90.0, β=90.0, and γ=90.0°	*a* = 169.4, *b* = 169.4, and *c* = 169.4 Å; α=90.0, β=90.0, and γ=90.0°	*a* = 169.1, *b* = 169.1, and *c* = 169.1 Å; α=90.0, β=90.0, and γ=90.0°	*a* = 168.4, *b* = 168.4,and *c* = 168.4 Å; α=90.0, β=90.0, and γ=90.0°	*a* = 169.3, *b* = 169.3, and *c* = 169.3 Å; α=90.0, β=90.0, and γ=90.0°
Resolution (Å)	28.78–2.75 (2.9–2.75)	42.35–2.7 (2.85–2.7)	20.66–2.4 (2.53–2.4)	37.65–3.2 (3.37–3.2)	29.92–2.9 (3.06–2.9)
*R*_pim_	0.035 (0.48)	0.028 (0.24)	0.045 (0.377)	0.052 (0.198)	0.077 (1.593)
*I*/σ*I*	16.2 (1.8)	24.0 (3.6)	9.8 (2.0)	10.1 (2.8)	7.0 (0.5)
Completeness (%)	99.9 (100.0)	99.9 (100.0)	97.4 (89.3)	99.8 (100.0)	99.9 (100.0)
Redundancy	12.5 (12.7)	12.6 (12.7)	3.5 (2.9)	13.6 (13.8)	15.6 (15.7)

**Refinement**					
Resolution (Å)	2.75	2.7	2.4	3.2	2.9
No. reflections	10743	11182	15351	6634	9030
*R*_work_/*R*_free_	0.212/0.242	0.215/0.251	0.233/0.264	0.220/0.246	0.249/0.266
No. atoms					
Protein	1158	1205	1216	1162	1165
Analog	87	87	87	36	31
Water	15	28	22	0	0
*B* factors					
Protein	56.0	34.1	59.1	64.4	57.4
Ligand/ion	68.8	42.7	68.9	80.2	72.6
Water	55.9	34.8	59.6	NA	NA
R.m.s. deviations					
Bond lengths (Å)	0.011	0.013	0.008	0.005	0.006
Bond angles	1.64°	1.75°	1.44°	1.31°	1.40°
PDB code	4JCZ	4JC7	4JRZ	4J7T	4J7Y

## RESULTS AND DISCUSSION

LTC4S is a highly specialized GSH *S*-transferase that converts LTA_4_ into the spasmogenic LTC_4_, an established asthma mediator. This enzyme is one of very few human integral membrane proteins whose structure has been solved at high resolution (15). Here, we combined mutational analysis with organic synthesis and crystallography to gain insights to the molecular mechanisms of LTC4S catalysis.

### 

#### 

##### Binding of Analog I–III Mimic of LTA_4_ and LTC_4_ Binding

The lipid substrate LTA_4_ is highly unstable (*t*½ ∼ 10 s) and cannot be used for co-crystallizations. To get a better picture of how the substrate and product bind at the active site and probe the role of Trp-116, we synthesized three mimics of an LTA_4_-GSH adduct, termed analogs I–III (Fig. 2). We solved the structures of wt LTC4S in complex with analogs I, II, and III to 2.75, 3.2, and 2.9 Å. Furthermore, the mutants W116A and W116F LTC4S structures were solved at 2.7 and 2.4 Å, respectively with analog I.

**FIGURE 3.**
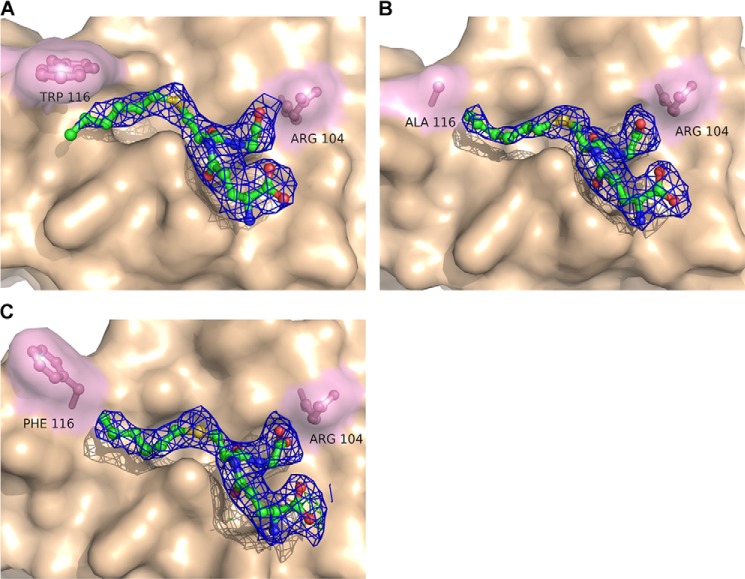
**2*F_o_* − *F_c_* maps contoured at 1σ of bound analog I with WT LTC4S (*A*), the mutant W116A (*B*), and the mutant W116F (*C*).**

**FIGURE 4.**
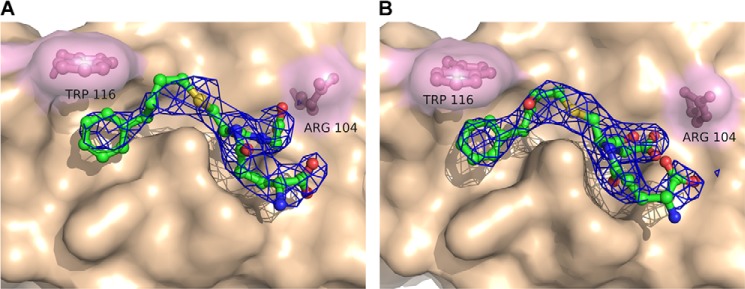
**2*F_o_* − *F_c_* maps contoured at 1σ of LTC4S with bound analog II (*A*) and analog III (*B*).**

The 2*F_o_* − *F_c_* maps with analogs I–III after refinement are shown in Figs. 3 and 4. The analogs were identified as strong positive residual density in the *F_o_* − *F_c_* maps after simulated annealing and initial refinement but before ligand introduction (data not shown). The phenyl ring in analogs II and III were added to increase the visibility of the acyl chain that was to some extent disordered in the analog I complex structure, as well as to stabilize its interactions between the protein and the acyl/phenyl moiety via π-stacking to Trp-116. The 2-hydroxy group in analog III was introduced to mimic the LTA_4_ epoxide opening in an attempt to shed light on the amino acid that would act as an acid during the catalytic reaction. In previous studies, Arg-31 was suggested to play that role (16). Analog III seems to result in tighter binding, as judged by the electron density and ability to inhibit enzyme activity; IC50 values for analog III were 0.4–0.8 mm as compared with 2.3–4.3 mm for analog I. A possible explanation for this might be the ability to form a weak hydrogen bond between the 2-hydroxy group of analog III to the carbonyl oxygen of Ala-20 in the adjacent monomer at ∼3.6 Å distance, an interaction that did not, however, provide new information about epoxide opening in LTC4S. The binding conformation, however, remains the same for all three analogs. Analog II showed a higher than expected IC_50_ value (>10 mm). The hydrophobic tails of all three analogs sit in the hydrophobic crevice created between two monomers and reach into the hydrophobic pocket under Trp-116 in which the ω-end of LTA_4_ is thought to bind and where a DDM molecule is bound in PDB code 2UUH. It has previously been suggested that this DDM molecule might be a good mimic for binding of LTA_4_. In light of the binding conformation of the analogs in the complex structures, we believe that the position of the aliphatic side chain of DDM represents LTA_4_ binding at an early step of catalysis where the thiolate anion attacks C6 of the epoxide ring, whereas the three analogs used here mimic the product as it is leaving the active site (see below).

##### Trp-116 Is Not Essential for Catalysis

We exchanged Trp-116 in LTC4S for an Ala or Phe residue and analyzed the effects on catalysis and kinetic parameters (Table 1). Considering the inherent variability that comes with enzyme assays with highly unstable lipid substrates, no major kinetic alterations were observed, thus demonstrating that Trp-116 is not essential for catalysis. The 3-fold increased LTA_4_ turnover of the W116F mutant may reflect a Phe configuration, which leaves the active site open to the lipid bilayer, thus facilitating product release (see below). The increased *K_m_* values of W116A/W116F for GSH are probably indirect effects of changes in LTA_4_ binding. The higher activity (two to four times) observed when LTA_4_ is varied as compared with when GSH is varied, is most likely due to a gradual loss of active enzyme as the concentration of GSH is reduced.

##### Trp-116 Helps Position the ω-End of LTA_4_

To further probe the role of Trp-116 for the positioning of LTA_4_ at the active site, we solved the structures of W116A and W116F LTC4S in complex with analog I, at 2.7 and 2.4 Å resolution, respectively. Comparing the omit map densities between the WT structure and the W116A mutant, it is clear that the acyl chain of analog I is less ordered (data not shown), which coincides well with previous proposals that the function of Trp-116 is to anchor the tail of LTA_4_. Hence, Trp-116 appears to aid in the positioning of the ω-end of LTA_4_ but this substrate-enzyme interaction seems to have very little, if any, impact on substrate turnover (Table 1). Of special note, in the structure of the mutant W116F, the phenyl ring has adopted a tilted conformation due to rotation at the α-β and β-γ carbon bonds (Fig. 3*C*). This rotamer opens the protein cavity toward the lipid bilayer. In addition, we have observed a similar twist of the natural Trp-116 in structures of LTC4S in complex with active site-directed inhibitors.^5^ Considering the position of Trp-116 in the middle of TM4, one could speculate that it shields the active site from interactions with the membrane bilayer or acts as a gate for substrate-product traffic between the protein and lipid phases.

##### The GSH Moieties of Analogs I–III Bind in a Tilted Conformation

**FIGURE 5.**
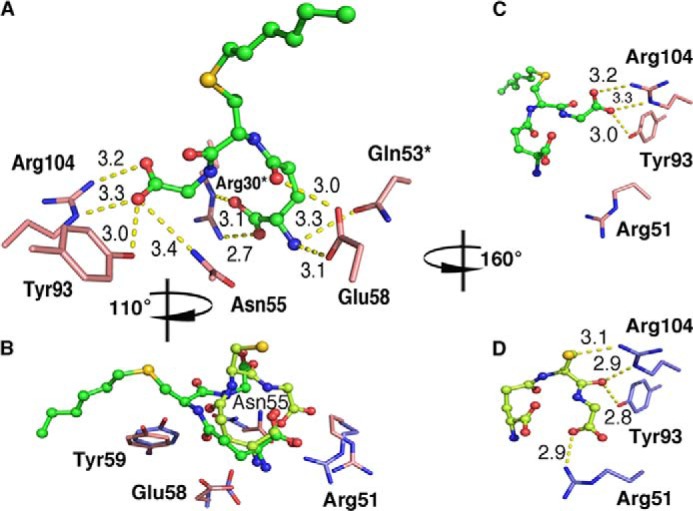
**Comparison between binding interactions of analog I (PDB code 4JCZ) and GSH (PDB code 2UUH) at the active site of LTC4S.** The overall coordinating network for analog I at the active site (*A*). Enzyme rearrangements at the active site with side chains in the complex structure with GSH depicted in *slate* and those with analog I in *salmon* (*B*). Residues that shift positional interactions between the GSH moiety of analog I (*C*) and GSH (*D*) are shown. Molecules are viewed from different perspectives, and rotations are indicated relative (*A*). *Yellow dashed lines* show distances. An *asterisk* indicates a residue from a neighboring monomer.

Examination of the active sites in the five analog complex structures reveals a shift in the binding configuration of the GSH part that is similar for all three analogs (Figs. 3 and 4). To detail binding interactions and analog positions, we used the structures of LTC4S and the mutant W116F in complex with analog I because they have the best interpretable electron densities. Binding of analog I at the active site of LTC4S is depicted in Fig. 5*A*. The coordinating residues are basically the same in our analog complex structures as compared with PDB code 2UUH with bound GSH. For both GSH and the GSH moiety of the analogs, the terminal carboxylates on the γ-glutamyl residue are coordinated by Arg30 and Gln53 from the adjacent monomer (Fig. 5*A*). Significant differences, however, are that some residues have changed their position and/or coordinate different parts of GSH as compared with the GSH moiety of the bound analogs. Thus, the side chains of Arg-51, Asn-55, Glu-58, and Tyr-59 are rearranged in the analog complex structures (Fig. 5*B*). In the structure with GSH, the thiol interacts with the catalytic Arg-104 to generate a thiolate anion and this interaction is lost in the analog structures. Furthermore, in the tilted conformation of the bound analogs, the catalytic Arg-104 together with Tyr-93, which coordinate the carbonyl of the cysteinylglycyl moiety of GSH in PDB code 2UUH, now coordinate the C terminus of GSH. Furthermore, the interaction with Arg-51 is lost, and this residue is then free to adopt a different rotamer (Fig. 5, *C* and *D*). Additional stabilization of the tilted GSH conformation is also likely provided by Asn-55, which no longer is close enough to interact with the carbonyl of the γ-glutamyl moiety of GSH and instead interacts with the C terminus of the GSH moiety of the analogs.

Unlike GSH, the analogs have hydrophobic side chains that interact with the enzyme. In PDB code 4JCZ, Tyr-59, Trp-116, Leu-115, and Ala-20 all line the hydrophobic pocket where the acyl chain of analog I and the phenyl rings of analogs II and III lie. As a result, the sulfur in the LTC4S analog I complex structure has shifted position by ∼6.8 Å as compared with its position in the GSH molecule of PDB code 2UUH. The sulfur in analog I in the W116A and W116F mutants shows a larger shift of ∼7.1 Å. The distance between Arg-31, proposed to be involved in catalysis (16), and the GSH sulfur is ∼7.2 Å in PDB code 2UUH. The corresponding distance in the structures with analogs I–III is ∼7.8 Å. The ω-end of the DDM molecule bound in PDB code 2UUH overlap from carbon-5 with the tails of analogs I–III. A slight difference is seen in the position of the ω-end in analog I in the three terminal carbons where in the WT structure they overlap with the DDM acyl chain as well as the phenyl rings of analogs II and III. In the W116A and W116F mutants, the three terminal carbons shift by ∼3.5 Å upwards toward the position of the Trp-116. This is most likely due to the larger space available in the absence of the tryptophan residue. In the W116F mutant, the phenylalanine is now pointing outwards to the solution/lipid bilayer and no longer functions as a “lid” for the hydrophobic pocket as seen with the Trp-116 in the WT structure.

##### Potential Mechanism for Product Release

Enzymes involved in fatty acid metabolism face a chemical problem in that lipids are poorly soluble in the cytoplasm or other aqueous compartments, which require special arrangements to allow substrate entry and product egress. Certain enzymes, for instance phospholipases and cyclooxygenases, cope with these problems through anchoring structures by which they get in close physical contact with the membrane compartment to make possible transport of lipids across the protein-membrane interphase (26, 27). For LTC4S, these mechanistic issues are even more complex. The entire protein is embedded in the lipid bilayer, and the enzyme handles two substrates with opposite chemical properties. GSH is a highly soluble tripeptide and must reach the GSH binding site located in between two monomers under the lipid bilayer (Fig. 1), whereas LTA_4_ is a hydrophobic fatty acid that also carries a highly reactive allylic epoxide, the binding site of which appears to be a superficial hydrophobic cleft below the membrane-protein interphase. Once the conjugation of GSH with LTA_4_ has occurred, the resulting product (LTC_4_) possesses both hydrophilic (GSH moiety) and hydrophobic (LTA_4_ moiety) properties (Fig. 2). Our data offer clues to the manner in which LTC4S manages these mechanistic problems.

**FIGURE 6.**
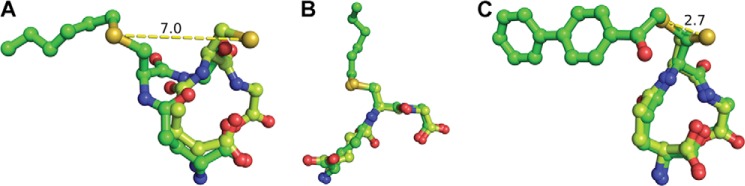
**Comparison between binding conformations of GSH analogs and native GSH in solved structures of GST.** *A*, binding of analog I (hexyl-GSH) and GSH in LTC4S from our structure PDB codes 4JCZ and 2UUH, a major shift in the position of the sulfur atom is indicated (7 Å). *B*, binding of hexyl-GSH and GSH in Δ class GST (adGSTD4-4) from *Anopheles dilus* (PDB codes 3F63 and 3G7I), *C*, binding of l-γ-glutamyl-*S*-(2-biphenyl-4-yl-2-oxoethyl)-l-cysteinylglycine and GSH to microsomal prostaglandin E synthase 1 (*mPGES-1*; PDB codes 4AL0 and 4AL1). GSH is depicted as a ball-and-stick diagram with a *light green* backbone, and analogs are shown with a *green* backbone. Nitrogens are colored *blue*, oxygens are *red*, and sulfur is shown in *yellow*.

The tilted configuration of the GSH moiety of the analogs is intriguing and is not observed when comparing structures of other GSTs having either GSH (PDB codes 1PKW and 3G7I), hexyl-GSH (PDB codes 2R3X, 3F63, 3F6D, 17GS, and 3PRN), glutathione sulfonic acid (PDB code 1GLP), or l-γ-glutamyl-3-sulfino-l-alanylglycine (PDB code 1YZX) bound (Fig. 6). In these structures, the GSH part seems to overlap regardless of which other GSH analog is used. Although the above proteins are all soluble, the overall reactions they perform are the same, *i.e.* conjugation of the hydrophilic GSH to a wide array of hydrophobic compounds (11, 28, 29). Recently, a high resolution structure of another MAPEG family member, microsomal prostaglandin E synthase 1 in complex with the analog l-γ-glutamyl-*S*-(2-biphenyl-4-yl-2-oxoethyl)-l-cysteinylglycine, was solved (17). In this structure, the GSH part of the analog overlaps with the GSH bound in the native structure (Fig. 6). This analog displays a very high degree of similarity with analogs II and III. Thus, it is tempting to speculate that the tilted conformation of the GSH moiety observed in our complex structures is a special feature of LTC4S and reflects specific facets of the catalytic mechanism.

**FIGURE 7.**
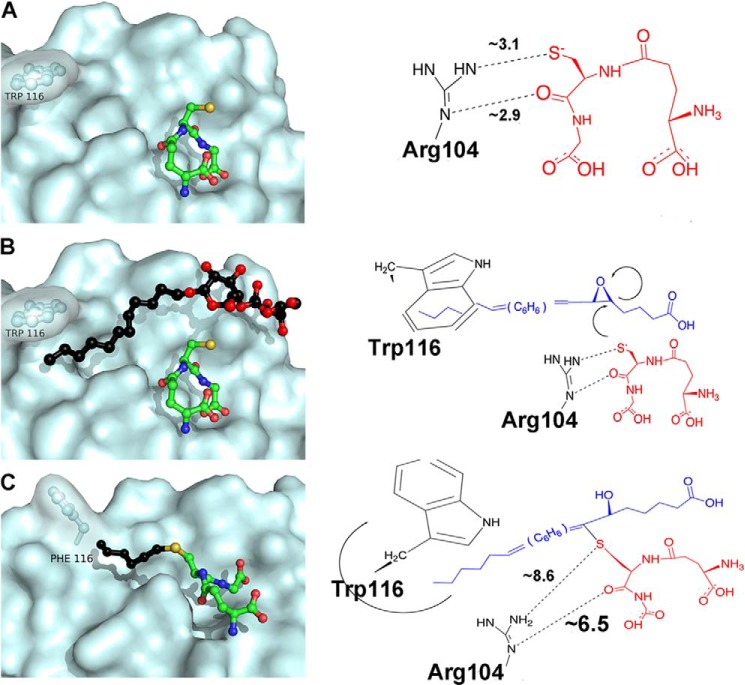
**Proposed mechanism for the conjugation of LTA_4_ with GSH and release of product from LTC4S.** *A*, the thiol of bound GSH (PDB code 2UUH) gets activated by Arg-104. *B*, LTA_4_ enters the active site with its ω-end shielded from the membrane by Trp-116, as indicated by the position of the aliphatic side chain of DDM (PDB code 2UUH). In the conjugation reaction, the thiolate attacks at C6 of the epoxide ring, presumably according to an SN2 mechanism. *C*, As LTA_4_ and GSH get coupled together the evolving product moves toward Trp-116, which rotates outwards exposing the product to the lipid bilayer and facilitating product release, as indicated by our structure of W116F in complex with analog I. Carbons of analog I extending from the sulfur are shown in as a *black* ball-and-stick diagram.

If one tries to integrate the results of our mutational analysis and structural information, one may speculate that after initial binding and activation of GSH, LTA_4_ slides into the hydrophobic crevice and reacts with the GSH thiolate at a point indicated by DDM binding (Fig. 7). The product then continues to slide toward Trp-116, as indicated by the tilted conformation of analogs I–III, which rotates around its side chain axis opening as a lid toward the lipid bilayer to allow product release (Fig. 7). Further studies are required to detail the discrete steps in the enzymatic conjugation of LTA_4_ with GSH, catalyzed by LTC4S.

##### Summary

To detail the role of Trp-116 for substrate binding and catalysis, we mutated this residue into an Ala or Phe residue and characterized the mutated enzymes. Together with crystal structures of wt and mutated LTC4S in complex with synthesized product analogs these data provide new insights to binding of substrates and product, identify a new conformation of the GSH moiety at the active site, and suggest a route for product release into the lipid bilayer, aided by Trp-116.

## Footnotes

5 D. Niegowski, A. Rinaldo-Matthis, and J. Z. Haeggström, unpublished results.

## Footnotes

4 The abbreviations used are:

LTA_4_: leukotriene A4, 5(*S*)-trans-5,6-oxido-7,9-trans-11,14-cis-eicosatetraenoic acid
LTC4S: leukotriene C_4_ synthase
LT: leukotriene
PDB: Protein Data Bank
DDM: *n*-dodecyl-β-d-maltopyranoside.
